# Needs and Expectations of Nurse‐Led Digital Support Among Parents of Children in Child Health Care

**DOI:** 10.1111/cch.70032

**Published:** 2025-02-14

**Authors:** Lotha Valan, Ulf Isaksson, Åsa Hörnsten

**Affiliations:** ^1^ Department of Nursing Umeå University Umeå Sweden

**Keywords:** child health care, digital support, nursing, parents

## Abstract

**Introduction:**

Sweden has an extensive national child health care programme (CHCP) implying that all parents are offered support to raise their children and support them for healthy development. The programme is today built on personal physical contacts and digital components unusual. Although the digital world could be frightening and insecure, it also has benefits, because it is not dependent on face‐to face meetings and is accessible more hours. In order to develop a digital channel to complement the CHPC, for the support of parents of children within child health care (CHC), parents' perspectives must be investigated. The aim of the study was therefore to describe parents' needs and expectations of digital support in the context of child health care.

**Methods:**

The study had a qualitative approach using workshop discussions with parents as data. The interview data were analysed using qualitative content analysis.

**Results:**

The main theme highlights that parents expected that a digital support channel would be something that might strengthen them towards independence concerning the care of their children, in a positive way. However, they also felt that they needed personal support and that relationships with other parents and the child health care nurse were significant and meaningful. Another parental desire that emerged was that a future digital channel would facilitate and simplify access to care and they suggested having both planned and urgent times available for parents to book. The digital channel was expected to make this possible and be a good complement to the physical contacts the traditional child health care currently offers.

**Discussion/Conclusions:**

The parents in this study believed that digital solutions could increase their parental power in relation to the care of their children. Examples were given as digital nurse‐led parent groups where parents with similar problems and experiences around their children could support each other and were expected to strengthen them over time. The parents stressed that a planned digital support channel also needs satisfactory solutions for both contact and response and have bookable digital meetings for both planned and urgent needs.

**Summary**

- Swedish child health care is not equally distributed due to distances.
- Child health care needs to be developed digitally.
- Parents' needs of child health care must be explored.
- A digital support channel might strengthen parents towards independence.
- Satisfactory solutions for both contact and response were urgent needs.

## 1 Background

### 1.1 Child Health and Support From Health Care

Parents can help their children to achieve the best possible health. The first years of life are crucial for the child's physical, intellectual, social and emotional development. Good family relations create bonds of trust and attachment between parents and their children. If it works successfully, healthy babies could grow into healthy children and later adolescents. However, some parents need support to help their children develop physically and emotionally (Raising Children Network (Australia) 2023). Support from health care—such as preventive health care visits or postnatal visits within 1 week, and parental groups—have been shown to promote and also lead to maintenance of good health in infants and their mothers, as well as promote individualized care and exclusive breastfeeding among women (WHO 2022a; Yonemoto, Nagai, and Mori 2021).

Quite few countries organize nurse‐led child health care. It is mainly nurse‐led, but team meetings with doctors are included in their programmes. These countries are currently Sweden, Norway, Portugal, England and Ireland, where the programmes are offered within primary health care. Most of the others countries have physician‐led programmes that highly focus on vaccinations and acute care, rehabilitation offered in private settings or within hospital care (The National Handbook for Child Health Services 2023; Bandiera, Ferreira, and Azevedo 2016; Staines et al. 2016; Håkansson et al. 2006). In a systematic review (Jeong, Franchett, and Yousafzai 2021), 67 studies were reviewed to produce recommendations on care interventions to support children, in this case, children under 3 years of age. The authors concluded that parents' knowledge about hygiene, infections and child nutrition needs to be increased. We believe that parents need much more than information about children's physical needs.

### 1.2 The Swedish Child Health Care Programme (CHCP)

Sweden has a national CHCP where all parents are offered support to raise their children and support for lifelong health. Child health care is voluntary and cost‐free, in which nearly 99% of Swedish families follow the programme delivered face‐to‐face via physical meetings and digital elements are unusual. Within the programme, children's health is promoted by identifying issues that have the potential to be problematic in relation to children's development, health and upgrowing environment (The National Handbook for Child Health Services 2023). The CHCP begins with a home visit by a child health nurse (CHN) to support and inform parents about the programme and to check the weight and health of the newborn child and the mother. Nurses who work within CHC are either district nurses or specialist nurses for children and young people. The programme continues with predetermined visits at the child health care centre at 1, 2, 3, 4, 5, 6, 8 and 10 months and 1, 3, 4 and 5 years. The visits focus on growth, the child's development process and support for parents in their role as the child's guardian. In Sweden, antenatal classes and postnatal group education are often offered but not nationally structured in the CHCP. This education is voluntary, offered individually and/or in groups, and differs between regions (cf. The National Handbook for Child Health Services 2023). Swedish child health care is intended to focus on the child, the parents and the rest of the family as well. Family‐focused care is today frequently pronounced as the assumption underlying nursing practice within child health care, which means to co‐create care together with the family, making the most of all competencies (Benzein and Hagberg 2017).

### 1.3 Parents' Needs for Support

The World Health Organization (WHO 2022b) has declared that parents themselves must be supported in feeling well to care for others; therefore, the well‐being of parents is a large part of the child's healthy growth and development. In Sweden, where mothers usually work, it is reported that several express insecurity and request continuous support and guidance from CHNs. Parents may also have their relatives some distance away, and the social network of friends and acquaintances is smaller due to social structure and the digital age (Ersson et al. 2021). A current literature review of 59 articles, synthesizing the qualitative evidence of women's experiences of postnatal care highlights an often unvoiced and unmet desire to acknowledge their care needs, especially their mental and emotional well‐being. They both expect and appreciate that postnatal health care resources primarily focus on their child and sometimes feel guilty about asking for help with their own needs. However, the synthesis concludes that most women need lots of support during the transition to motherhood and appreciate professional help with emotional and psychosocial concerns (Sacks et al. 2022). The need for targeted parental interventions to support children's health is highlighted in a guideline by WHO (2020), and it also states that there is a lack of research on caregivers other than mothers. It further requests care interventions that can support children's behavioural and socio‐emotional development.

### 1.4 Time for Development and Digital Solutions Within CHCC

Even if the child health care in Sweden and its national programme is well visited (99%) despite long distances, it has differences in supply. For example, in rural areas, parental support is not always provided in groups due to the distance to child health care facilities. It is not equally distributed across the country and therefore needs to develop (Tell, Magnusson, and Lindfors 2011) and also in digital forms. Besides the predetermined visits and frequently offered thematic parental group education, more is needed to satisfy today's parents because many (about 60%) are not attending these. Ersson et al. (2021) conclude that even if parental groups as education and support is a good approach, it also calls for more forms, such as digital support. One example is Pakarinen et al. (2018), who designed a digital intervention for parents of 4‐year‐old children. The intervention enabled support for individual families' health knowledge needs and has been implemented within Finnish child health clinics. An Australian study by Muscat et al. (2020) offered practical insights into the possibility of embedding a digital health skill intervention in parental groups. These parents were informed about the programme, and participation was offered to the parents already during the first home visit. The study showed that this intervention could increase their health competence (Muscat et al. 2020). Valan et al. (2018) also concluded that child health care professionals should be more educated and provide digital solutions. From a study by Bianco et al. (2013) where parents' health‐related searches on the Internet were investigated, it was concluded that personnel within health and medical services should offer education about internet searches to the parents, which could involve how to assess information found critically and how to navigate to achieve high‐quality information. A Norwegian study (Skranes et al. 2014) reported that mothers found the Internet valuable for advice about their children and used it daily. In addition, they often sought self‐care advice on the Internet if their child was ill or was suspected of being ill. They also reported that the Internet was not always easy to navigate for parents, and they may need support. Therefore, facilitating people's use of and searches on the Internet and promoting eHealth is important and is suggested to be a task for health care professionals. In a systematic review by Kubb and Foran (2020), which aimed to examine the prevalence and associated variables for searching health information online for parents and children, they identified gaps in Internet searching. They demonstrated how parents were influenced in their health behaviour and concluded that parents should be supported in the right way to use the web for health purposes more effectively.

Because parents of today increasingly seek answers regarding children's health problems on parental forums, Farrell (2018) examined the accuracy of online discussion forums and if the information agreed with information in evidence‐based, point‐of‐care resources. The findings showed that only a small amount of the information was inaccurate or misleading. However, this does not mean that all information should be trusted, something many parents also are aware of.

In order to use parents and their competencies as a starting point to learn more about their needs and expectations of content and forms of digital support in the context of child health care, we decided to perform workshops where parents met with their CHNs, an IT expert and researchers to discuss possible technical solutions and forms, individual and in groups as well as the topics/areas or situations that they felt they expected to benefit from by discussing and meeting the CHN as well as other parents digitally within the context of child health care.

### 1.5 Aim

This study aimed to describe parents' needs and expectations of digital support in the context of child health care.

## 2 Method

### 2.1 Context, Settings and Participants

The study took place at child health care centres, which in Sweden are a part of the assignment of primary health care. These centres are often located within or close to the local health care centres. Because we wanted at least two CHNs to be involved, we chose to include two child health care centres from two socio‐demographical comparable health care centres in mid‐Sweden. Within the actual catchment area, seven health care centres were run. Of these, one was very large and located in the centre area, thereby excluded. Two were small and located in very rural areas. Of the remaining four health care centres, all were of similar size and located outside the city centre. We finally chose the two most similar regarding sociodemographic structure with an industrial area, a village centre and also a sparsely populated rural area, where attendance often is lower. The two selected health centres were also similar in terms of child health care and families' participation in child health care visits. In total, about 5000 and 6400 persons were registered at the respective health care centres.

The participants who offered to participate were parents of children from the two health care centres. Parents were consequently invited to participate in digital workshops highlighting their needs and expectations. Posters about the study were placed in the waiting room of the child health care clinic. The poster contained a description of the study, its aim, information about the assignment and whom the parent should contact for more information or participate in the workshops. They were referred to the child health care nurse (CHN) for more information if they were interested and for registration in the study.

Twenty parents were initially interested, where 12 parents finally agreed to participate. Eight parents later declined because they did not accept being recorded. The 12 parents that accepted participation were all mothers; four were first‐time parents, and the others had two to three children. They were invited to an initial workshop (Table 1) where only six parents could participate on that date. The other six parents were invited to another date, where five participated. The last parent among the 12 had a single workshop because she really wanted to express her experience as she was living in a rural area. Then, on request, a second workshop session was conducted for complementary questions and a deepened discussion because some parents (*n* = 5) wanted to express their opinions further (Table 1).

**TABLE 1:** Overview of the workshop sessions (*n* = 2) and participating parents (*n* = 12).

Initial workshop (session)	Workshop participants (no of parents)	Repeated workshop (session)	Workshop participants (no of parents)
I	6	II	5
	5		
	1		
	In total 12		In total 5

### 2.2 Workshops

Performing the workshops was a first step in developing a user‐centred intervention where stakeholders, that is, parents (*n* = 12), but also two CHNs, two researchers in nursing, as well as one person with expert knowledge in informatics and computer science, were invited to discuss and thereby possibly also influence the design of the forthcoming intervention. The role of the CHNs in the workshop was to moderate the discussions and to reflect the parents' suggestions against the existing child health programme. The role of the informatics and computer expert was to reflect about if the parents' ideas could be possible within existing child health computer systems and security regulations. The role of the researchers was to observe the sessions and to highlight new questions they found important. The participating parents had experience with the traditional CHCP of home visits, team and CHN led visits. The participants were free to highlight their questions and wishes during the workshops, but questions planned on forehand to discuss during each workshop were the following:

- What are the wishes and needs regarding the structure and content of a digital channel?
- What needs of support, in addition to ordinary child health care activities, do parents have today?
- How could CHC be developed and improved using digital technology to be more supportive of parents?
- What development opportunities exist that are attractive to parents within the existing framework for technology use, ethical principles and privacy legislation?
- How do we proceed? What should we focus on, and what seems unreasonable?

### 2.3 Analysis

The recorded workshop discussions were transcribed into text (72 pages, Times New Roman, text size 12 and 1.5 row/line spacing) by the first author. The text was analysed using inductive qualitative content analysis described by Graneheim and Lundman (2004) and Graneheim, Lindgren, and Lundman (2017). Qualitative content analysis was chosen because it has the flexibility to gain a detailed and broad understanding of the collected data. Initially, the transcribed texts were read thoroughly to grasp a meaningful understanding, which also was discussed in the research group. After that, the transcriptions were analysed line by line by the first author, where meaning units responding to the aim were identified and marked. The meaning units were then condensed when needed, that is, made shorter but with its core message remained. The condensed meaning units were thereafter abstracted and coded, meaning given shorter labels that described their content. The codes were then compared on similarities and dissimilarities and thereafter grouped, with input from the whole research group. Next, the codes were interpreted on a higher level and sorted into subthemes and themes, and lastly, a main theme could be identified as a red thread going through the other themes on various levels. This analytic process should not be seen as a linear process. Instead, it involves going back and forth between original text, abstractions and interpretations in a complex analysis and where trustworthiness is strived for in each step. Transcription, choosing appropriate meaning units and coding them close to the text, abstraction and interpretation of themes in various levels were discussed in the research group in which consensus was strived for (c.f. Graneheim and Lundman 2004; Graneheim, Lindgren, and Lundman 2017).

### 2.4 Ethical Considerations

The study has followed the general ethical principles for research, that is, informed consent, confidentiality and the right to withdraw from participation. We have also got permission from the Swedish Ethical Research Board (Dnr 2021–00412) and from managers of the primary health care centres.

## 3 Result

The sample consisted of 12 mothers (M1–M12) in ages between 26 and 38 years. Their education varied between highschool and PhD level. All lived 4–29 km from the child health care centre. All were cohabitating with the father of the registered child, and the families had one to four children.

The analysis answering the aim to describe parents' needs and expectations of digital support in the context of child health care resulted in one main theme built up by four themes with two to three subthemes each (Table 2). The result is also described in the text below, with subthemes in italics, exemplified with quotations from the original text.

**TABLE 2:** Parents' needs and expectations of digital support in the context of child health care.

Main theme: Becoming empowered in parenthood but still in need of support and meaningful relationships	
Themes	Subthemes
Improving parental confidence	Increased knowledge Important confirmation Reduced worries
Creating longer term relationships	Feelings of safety Family‐like treatment Continued support
Improving accessibility	Easy access to health care Reassured contact and response
Strengthening independence	Increased parental power Shared experiences with other parents Reduced loneliness and weakness

## 4 Becoming Empowered in Parenthood but Still in Need of Support and Meaningful Relationships

The main theme highlights that parents expected that the digital support channel would be positive and something that could strengthen them towards independence. However, they also felt that they needed support and that relationships with other parents and the child health care nurse were meaningful. The digital channel was expected to allow bridging these aspects and could become a good complement to the physical contacts they currently had.

### 4.1 Improving Parental Confidence

The digital channel was expected to improve their parental confidence. It could increase their knowledge because they could meet more frequently, take advantage of other parent's experiences and get input from their child's health nurse. These meetings were also expected to discuss smaller and larger issues and give them essential confirmation that they had managed their children's developmental or health issues right. Within this theme, it was also highlighted that a digital channel with easy access could reduce parental worries, strengthening them for the future and their confidence to handle their children's potential problems or health threats in time.

#### 4.1.1 Increased Knowledge

Increased knowledge was expected to improve the parents' confidence. In a digital channel, they would get answers to questions when they needed. Most parents described that they mainly used Google to search for internet information if they wondered something about their children's health. They often found the Internet helpful but expressed that it could be disappointing, for example, when the answers varied on various websites. Many described that they had been in more minor incidents with their children and did not know if it was severe.

… you should be able to avoid constantly seeking acute care [if it is not necessary], but if you can get a first, you know … message or … of someone who knows … . (M 2)

Contacting digital support and quickly getting an assessment and some information on what to do was suggested to increase their knowledge of the situation and prepare them to handle similar situations. Many parents also felt that a digital support channel would make it easier to get personalized knowledge by questioning professional nurses who were familiar with their child and could advise them in the particular situation.

#### 4.1.2 Important Confirmation

Several parents suggested that a digital channel that included requested thematic meetings or targeted forums with other parents and a CHN could improve their confidence and confirm that they, in various situations, had thought right and made the right choice with their children's best in mind, for example around continuing or stopping breastfeeding or how to manage their children's sleeping problems.

But if there is some topic you want to learn about, digital support would be good, and then it would be fine in groups of other interested parents so you can discuss with them … kind of like targeted forums … . (M 11)

Such thematic meetings could imply requested topics decided on forehand, with a nurse present that could be involved in the discussions and confirm their various opinions, adjust them or at least recommend other ways to solve a situation if needed. Most importantly, they could find support and confirmation from other parents who had experienced the same.

#### 4.1.3 Reduced Worries

Some parents said too many choices and different Internet search answers worried them. They expected that a digital channel where their CHN was involved could reduce their worries, expand their understanding, and give them courage to act. They described it as essential to reach digital child health care relatively quickly when they were worried or needed answers, even if it later was understood that it was not an acute problem.

Child health care is much more than acute care. You can have problems like sleeping and things that you can wait with … to just know that you do not call 1177 [Swedish National Telephone Advice] about it, but it is more than weighing and measuring at the Child Health Care clinic as well … . It supports too. (M 5)

The parents expressed that their needs and worries were the same, but a digital support channel would develop CHC and what they previously had been offered. However, some expressed that CHC felt a little outcompeted by the internet development. They suggested that CHC could take advantage of the Internet when developing the digital channel by responding more personalized to the parent's needs and thereby reducing worries, increasing knowledge and confirming that they are doing a good job in their parental role, something that would empower them for the future.

### 4.2 Creating Longer Term Relationships

Over time, the frequent contacts and relationships with CHN were experienced as creating long‐term relationships already today. However, a digital channel could imply even more frequent contact, continuing after the child's first year of age, and could be more tailored to the parent's support needs. The parents expressed that using a digital channel would feel close and related, not at a distance, which is usual on the Internet because the nurses already knew the families and had an overview of the children's childhood and upbringing. Feelings of safety could be strengthened, and family‐like support could be even more familiar and continue over time, creating even longer term relationships.

#### 4.2.1 Feelings of Safety

Even if a digital channel uses the Internet, it was expected to make them feel safer and more secure than making regular Google searches or sometimes even visiting the CHC clinic. Digital support felt safer because it was arranged professionally by public health care. The parents also believed that it would make it easier, and they would feel safer participating from their own home, communicating in a less stressful environment and still receiving support via a mobile phone or computer. To avoid taking a feverish or sick child out, dressing the child in outfits and driving to the health care centre to get an assessment would make life easier. Based on their experiences and depending on the necessary equipment, assessments were almost always seen as possible to make digital via video. One of the mothers said that if the digital support had been developed when her child was younger, she would have used it as a breastfeeding support to get the feeling of increased security.

I expect it would be improved [the support] with the help of digital technology. If you have breastfeeding problems, I could sit at home in peace while being on the phone or the computer, and the nurse could cheer and confirm that yes, that looks good, or when you got sores, saying try doing this instead …. (M 10)

#### 4.2.2 Family‐Like Treatment

The relationship between the nurse at CHC and families was also today experienced as more familiar and permissive than other forms of health care. The parents expressed that they appreciated the familiarity in the relationship between the CHNs and themselves. The relationship between the parents and the CHN was expected to be facilitated further through a digital channel. They also felt they could get answers from the CHN that suited them and their children better than from other telephone advisories where the health professional had never met their child.

To have, like our nurse who have the background, the conversation becomes more personal and more detailed and precisely, what is happening just now …. (M 9)

They also pointed out that their relationships with other parents, having similar experiences with their children, had become more familiar. They expected it to be developed even better through digital group support sessions, where parents living rural with long distances could participate.

#### 4.2.3 Continued Support

The long‐term relationship with the CHN was seen as beneficial. The parents expressed that it could expand even further with help from digital techniques and thereby become more supportive because new problems can arise between regular annual or biannual visits. Having a long‐term relationship between the parents and their CHN was perceived as allowing continued discussions of a recurring problem without starting over with explanations.

It's easier to go via the child health care, I think, because they know who my boy is and everything else you've talked to the nurse about, i.e. regarding him …. (M 6)

The parents described that when they visited physicians at the health care centre, they had to tell them everything—from the beginning—to make them understand the problem. On the other hand, through a digital support channel, they could send an email or have a video chat and tell the CHN that the child has this problem again and needs treatment. With continued support, a digital channel would facilitate continuing the conversation where it ended last time and an easier way to follow up and get assessment and information about what to do.

### 4.3 Improving Accessibility

A digital support channel within CHC was, by many parents, described first and formerly as improving accessibility. Easier access through digital contacts, meetings and support within CHC was seen as facilitating parenthood and could avoid parent's contact with doctors in primary or acute care when unnecessary. It was also expected to reassure contacts and respond to their questions when they needed support and between planned meetings.

#### 4.3.1 Easy Access to Health Care

Parents expressed the need for improved access to health care in general, particularly to the primary health care centre and CHC. Some had experienced that, for example, after a weekend with a child with a fever, being worried about calling the primary health care centre on Monday morning and getting the information that the dial‐up service already is booked. They also gave examples of being busy at work and having difficulty waiting to be dialled but still having important questions or other needs. A digital channel was expected to make it possible to be away, be out of town, and still be able to book a meeting and get advice and counselling about important issues. The parents gave many other examples of where a digital support channel within CHC would facilitate their lives. For some who live in rural areas, digital meetings could save miles and time. If something quite acute happened and they needed a fast answer, they expected to get support via a digital meeting in a few minutes.

We are living rural in a village on a distant island, and instead of taking the boat into the city, you rather google … Say that my child wakes up and is red all over the body, and now during the winter, when the boat goes ashore at 07.00 in the morning … Today I can book an appointment for a meeting the next day at the CHC at 09.30 … . However, I must wait until three pm before the boat returns to the island, the only trip that goes now during the winter … . So instead, if I could book a digital meeting [through video] … I can still meet her at 9.30 and receive advice … . Then I can call my brother [in the city], and he buys what you prescribe … and deliver it to the boat, and in the afternoon, I can start lubricating and not use the time for travel and waiting the whole day. (M 12)

Easy access to these digital meetings was desirable because many parents felt that CHC, as of today, had limited availability. As a parent said, they got a letter with a booked time. If that was not suitable, the parent had to call the health care centre at a special particular hour when they had telephone time or in a telephone system that redialled them later during the day or the day after, which was expressed as unsatisfactory.

#### 4.3.2 Reassured Contact and Response

Parents expected the digital support to reassure contacts and response could facilitate them, particularly when their children were sick. The parents expressed that it would be valuable to get an initial digital contact, including an assessment by a professional nurse, and within that support, be able to ask questions and get treatment advice, or if it seemed to be acute and they should go to the hospital. The parents accentuated that the planned digital support channel needed updated accessibility solutions for contact and response. To have new and acute times to book for parents and options to rebook times were suggested. Another expectation was an easy solution for contact by email. Something that was requested was that the parents could send a photo of a wound, a rash or something else and receive an answer within a few hours. As some parents expressed, having a response in an email with the photo they sent with information if they should come into the health care centre and get an answer if the problem should be acutely treated or not would make things easier for the whole family and probably save resources for the health care.

… it would be great if you could send an SMS or an email in addition to or between the visits. It's really, really worth it! Because otherwise I must call the health centre, book a re‐dial, ask for an appointment, wait days, then take my child, pack all stuff in the car, and drive …. (M 8)

### 4.4 Strengthening Independence

The parents felt that a digital support channel with easy access could strengthen their independence over time. Their parental power could increase, as well as relations with other parents and shared experiences with them could do the same. Overall, a digital support channel was expected to reduce loneliness and feelings of weakness in situations they today must handle alone.

#### 4.4.1 Increased Parental Power

In order to become empowered, the parents expressed that they would like to be seen as a family unit and not only as parents just attached to the child. Several parents also expressed needing support as a couple in their parenting role. The parents expressed that, on the one hand, a digital solution could pose difficulties in embracing both parents and all family members in meetings. However, on the other hand, physical meetings were described as having the same problem.

But if it was something you wanted to learn about, digital support would be good, and then it would be good to have more parents so you can discuss … sort of like targeted forums …. (M 1)

The parents expressed that digital support should involve at least both parents, sometimes divorced, sometimes in different geographical locations, at home, at work and so forth. Because the family can be a small unit with particular problems and needs, the support was expected to meet those. A diverse amount of suggestions on forms of and topics of digital group meetings to increase their parental power was given. It included topics and opinions not taken for given by parents, even if they experienced that the CHN often have a clear opinion about them. Such topics put forward were, for example, the pros and cons of breastfeeding, tackling children's and parents' weight problems, children sleeping in parents' beds, and traditional subjects such as opinions about introducing food and taste portions related to allergies. If discussed from several points of view, such discussions could increase parental power to decide by themselves.

#### 4.4.2 Shared Experiences With Other Parents

Sharing experiences with other parents was seen as strengthening independence. Digital meetings with other parents could imply discussions that provide understanding among each other. Examples were given by parents who had been traumatized by having a child previously involved in some accident or acute incident. The parents seemed to come closer to each other by sharing such dramatic experiences, and they suggested that such free discussions could be important when planning digital support for parents. Parental groups with no topic decided on forehand were therefore also suggested where they could meet other parents with similar needs, let the parents discuss more personal questions, and have the nurse only as a resource in the group.

Meetings more like a Zoom meeting would have been very good … You could have meetings about different things, like the one about eczema and lice … Those that are planned and those who are open, like a free discussion for questions in general around everything you like … it would have been great. (M 3)

A recurring group meeting at the same time each week was suggested, and all parents seemed to appreciate that idea. That time could be an opportunity to get to know other parents and discuss different self‐chosen topics, strengthening independence.

I think it sounds very interesting with these ideas of digital Wednesday coffee meetings because if you have a question or some topic you think about, then you know that on Wednesday you have the opportunity to … lift this question face to face, even if it is digital. (M 7)

Some parents who described themselves as shy saw an opportunity to participate and listen but not necessarily need to talk a lot themselves. Others saw it as a forum to raise topics that had come up during the weak and wanted a response. However, even in these parent‐centred meetings, the parents saw it as necessary that the nurse was present for the morale and tone of the meeting.

#### 4.4.3 Reduced Loneliness and Weakness

Some parents meant that a nurse‐led digital support group for parents could reduce loneliness and feelings of weakness and instead strengthen them. Some had previously participated in parental forums and chats, and most participants used various kinds of social media but had also experienced the hatred that often comes with it.

… in these chat groups, it will be easy to … eh … it feels like a pretty hard climate … or you might interpret it as more of intrusion … . Although it may be well meant that … you only see the words, you do not see the feelings behind …. (M 2)

Several expressed it as unpleasant and had feelings of not always living up to the parenting standards when visiting such public sites. They thought that with a CHN present who would moderate the discussions, the interactions and climate in the group could be better. All the parents thought that such digital support would be strengthening. Meeting with other parents in the same situation was also expressed as important to reduce loneliness. Even if the meeting is digital, it was seen as easier to talk to parents in a similar social context with children of the same age as their own.

… when they come to this two‐year‐old age, there are completely new challenges that you would like to discuss with other parents. So, have some kind of [age‐specific] parent group for it …. (M 4)

## 5 Discussion

Our study explored parents' needs and expectations regarding digital support within child health care, with the main theme being ‘Becoming empowered in parenthood but still in need of support and meaningful relationships’. The findings suggest that although digital support is appreciated, the relationship between parents, CHNs and other parents remains crucial. Therefore, digital support should complement physical interactions, providing a similar sense of connection. Nonetheless, parents expect significant benefits from digital support systems.

One key finding is that parents believe digital support can boost their confidence in parenting. Many suggested that thematic digital meetings or forums involving other parents and CHNs could enhance their knowledge, validating their choices for their children's well‐being. This reassurance could reduce their anxiety, helping them feel more confident. Although these parents did not face special challenges, Armstrong‐Heimsoth et al. (2017) emphasize that all parents could benefit from health literacy training. Their study showed that increased health literacy improved confidence in finding credible health information online, aligning with our findings. Parents, particularly mothers in our study, expressed challenges in finding reliable evidence on topics like breastfeeding or formula feeding. Digital group discussions that blend evidence‐based information with parents' shared experiences could alleviate these concerns and build confidence over time.

Another significant expectation was that digital channels could foster long‐term relationships. Parents valued continuity with CHNs, especially as visits become less frequent after the first year of life (cf. The National Handbook for Child Health Services 2023). Digital visits or group meetings between these in‐person check‐ups could strengthen bonds between parents and CHNs, as well as with other parents, offering a sense of safety and security. Jansson et al. (1998) noted that long‐term continuity with a single CHN fosters trust, making parents more receptive to advice. Similarly, our participants expressed that this continuity builds familiarity, easing interactions and making advice more meaningful. As Ersson et al. (2021) noted, today's social structures—with fewer face‐to‐face interactions with friends and relatives—make the CHN relationship even more crucial for support and guidance.

Improved accessibility was another prominent need. Parents believed digital support could enhance the existing child health programme, but only if it is easily accessible. This aligns with research by Walker et al. (2017) and Cowey and Potts (2018), who emphasize the importance of simple, flexible, and accessible digital health solutions. Although digital tools are often seen as easier for younger generations, parents believed such technology should also be user‐friendly for CHNs, who are often older.

Parents suggested that digital meetings could be integrated into CHNs' daily schedules, offering short, open sessions for parents' questions (cf. Jafree et al. 2021). Reliable websites recommended by CHNs could provide 24‐h access to credible information, though it is crucial that CHNs are knowledgeable about these sites and that they are user‐friendly across diverse social contexts. Walker et al. (2017) call for collaboration with ‘mother‐friendly’ websites, suggesting that such resources must be designed with the target group's needs in mind. For example, parents in our study highlighted the importance of receiving support on topics like breastfeeding from the comfort of their own home via digital connections.

Finally, parents expressed that digital support could empower them and reduce parental stress. Albright Bode et al. (2016) found that knowledge increases parental independence and reduces stress, as parents are better equipped to care for children. In our study, parents expected that a digital channel offering stepwise learning could strengthen their independence and confidence over time. This echoes findings from Jafree et al. (2021), where Pakistani women who received digital support reported greater independence and improved family health through increased knowledge.

Reducing feelings of loneliness was another anticipated benefit of digital support. Many participants lived in rural areas, far from social centres and felt isolated during parental leave. Digital group meetings could help alleviate this isolation. Ezran et al. (2021) and Tell, Magnusson, and Lindfors (2011) also point out that rural parents often face greater challenges accessing health care, which could be mitigated through digital solutions. Video calls and online parent groups were suggested to overcome the barriers of distance and transportation.

In terms of design, parents wanted both scheduled and urgent digital meetings, with the ability to quickly contact a CHN for advice. This could save time and resources, as CHNs could help parents determine if emergency care is needed, potentially reducing unnecessary visits (cf. Valan et al. 2018). Overall, parents expected that a digital support channel would empower them by offering both peer and professional support, ultimately increasing their confidence and reducing anxiety.

### 5.1 Methodological Considerations

Although the offer to participate in the study went to all parents, only 12 mothers agreed to participate in the workshop, a weakness that may be due to the parental leave outtake in Sweden. Eight parents decided not to participant due to recording. For future, one workshop could have been offered without recording and instead taking notes during conversations. A larger sample might have given additional aspects of what a digital supplement needs. However, the groups were considered sufficiently large for all participants to dare to share their opinions and all are quoted in the result. One parent participated with the CHN in a single ‘workshop’ or interview. This was not planned in the study design, but this parent could only participate on that date, and her views were seen as important because she lived very remote.

## 6 Conclusion

In summary, the parents were very positive about the opportunities a digital supplement in child health care could provide. They expressed that a digital support would be a way for them to become empowered and feel continually supported in CHC. The parents expected that their safety could increase through nurse‐led digital meetings and that increased knowledge and confirmation from the CHN would reduce anxiety and increase their confidence. The parents gave many examples of where a digital support channel within CHC would facilitate their lives if it had easy access. Families living in rural areas could save miles and time, among many other benefits as reducing loneliness from digital support and parental groups. In conclusion, Swedish child health care and the CHCP could use these findings to find motives for modernization, focusing on increased digital meetings.

## Author Contributions

We have used the CRediT taxonomy for reporting the author contributions and the authors Lotha Valan (LV), Ulf Isaksson (UI) and Åsa Hörnsten (AH) have been involved as below. Conceptualization: LV, UI, AH. Data curation: LV, UI. Formal analysis: LV, AH. Funding acquisition: LV, AH. Investigation: LV, UI, AH. Methodology: LV, UI, AH. Project administration: LV. Resources: LV, UI, AH. Software: LV. Supervision: UI, AH. Validation: LV, UI, AH. Visualization: LV, UI, AH. Writing – original draft: LV, UI, AH. Writing–reviewing and editing: LV, UI, AH.

## Ethics Statement

The study has followed the general ethical principles for research, that is, informed consent, confidentiality and the right to withdraw from participation. We have also got permission from the Swedish Ethical Research Board and from managers of the primary health care centres.

## Conflicts of Interest

The authors declare no conflicts of interest.

## Data Availability

The authors declare that data supporting the findings of this study are available in the paper.
